# The effects of distributed leadership on teaching innovation in Shanghai, China: the mediating roles of teacher autonomy, teacher collaboration, and teacher self-efficacy

**DOI:** 10.3389/fpsyg.2025.1562838

**Published:** 2025-07-14

**Authors:** Jing Gong

**Affiliations:** School of Education, Wenzhou University, Wenzhou, China

**Keywords:** distributed leadership, teacher collaboration, teacher autonomy, teacher self-efficacy, teaching innovation, self-determination theory, conservation of resources theory

## Abstract

**Introduction:**

Teaching innovation (TI) is crucial with respect to efforts to cultivate innovative talent and enhance national competitiveness. To promote TI, the Chinese government has issued policy documents that have emphasized the need to overcome the traditional management model, which focuses on the “principal's individual heroism,” and to encourage schools to implement distributed leadership (DL) with the goal of achieving multiparty cogovernance. However, few studies have investigated whether and how the implementation of DL in the centralized and bureaucratic management models used in China can foster TI among teachers.

**Methods:**

To address this gap, this study examines 3,976 teachers working at the lower secondary level in Shanghai, China, on the basis of data obtained from the 2018 Teaching and Learning International Survey (TALIS) dataset; in this context, a structural equation model (SEM) is used to compare a parallel mediation model constructed by reference to self-determination theory (SDT) with a chain mediation model constructed through the integration of SDT with conservation of resources theory (COR).

**Results:**

The results of this research reveal the superiority of the chain mediation model, according to which two independent mediation paths pertaining to teacher collaboration (TC) and teacher self- efficacy (TSE), as well as the two chain mediation paths of TC → TSE and teacher autonomy (TA) → TSE, are significant with respect to the relationship between DL and TI.

**Discussion:**

Theoretically, this study clarifies the mechanism underlying the influence of DL on TI in collectivist cultures; practically, it provides a reference for management practice for lower secondary school teachers with respect to TI.

## Introduction

Teaching innovation (TI) serves as a foundation for efforts to nurture innovative talent and enhance national competitiveness. In 2025, China released the “Outline of the Plan for Building an Education Power (2024-2035),” which highlighted the importance of advancing education modernization as a pivotal approach to the task of comprehensively deepening overall educational reform. Distributed leadership (DL), which focuses on modernizing school governance in China, aims to decrease teachers' sense of being controlled and to elicit enthusiasm for TI and organizational engagement through the delegation of leadership authority. In China, which is largely characterized by a bureaucratic management model, the question of whether the implementation of DL, which is closely associated with Western neoliberalism (MacBeath, 2005), can effectively inspire TI is worth investigating. The negative perspective on this topic notes that in Chinese educational organizations, bureaucratic centralized management is a common structural model used for organizations. Under this system, individual power and responsibility are typically embodied in “formal leadership positions,” which feature clearly defined responsibilities and rights. When an organization implements DL and engages informal leaders in school management, the existing responsibility and authority system is disrupted. Teachers who are not granted formal leadership positions are required to bear more leadership responsibilities and authorities. This situation can give rise to ambiguity, conflict, and imbalance with respect to teachers' roles, thereby not only undermining teachers' organizational citizenship behavior but also negatively affecting the quality of school teaching (Harris, 2003). In contrast, the positive perspective on this topic focuses on the balance between centralization and decentralization in organizations. Scholars who have adopted this perspective have tended to view decentralization as a “resource reward” rather than a “role conflict” due to their belief that overcoming the issue of “decision-making isolation” within the bureaucratic system and relying on a fluid empowerment network can promote professional wisdom and TI among teachers (Teng et al., 2024; Unsworth et al., 2005). This controversy offers space for this study to investigate the relationship between DL and TI in the Chinese context.

DL is an external influence on TI. According to self-determination theory (SDT), if external motivation can satisfy individuals' three basic psychological needs, namely, the needs for autonomy, competence, and relatedness, it can stimulate TI among teachers (Deci and Ryan, 2000; Amabile and Pratt, 2016). Previous studies have extensively examined the crucial roles played by these three psychological needs in the process of transforming extrinsic motivation into innovative behavior, and these three needs have often been indicated to influence people's feelings and behaviors in parallel relationships (Ahn et al., 2021; Chong et al., 2021). However, some evidence has suggested that the satisfaction of certain need(s) promotes the satisfaction of other(s) (Granjo et al., 2020; Cai and Tang, 2022). The quality and extent of different motivations are inevitably influenced by social interactions and living environments (Weinstein and DeHaan, 2014). During the critical period of efforts to promote education modernization in China, schools integrated the management concept of democratic cogovernance into the traditional bureaucratic management structure with the goal of establishing a balance between centralization and decentralization in this context. On the one hand, under the influence of traditional collectivist cultures, teachers prioritize collective interests. They adhere to clear hierarchical relationships and role divisions within the bureaucratic system and promote the achievement of collective goals through group connections and teamwork among teachers. This approach motivates teachers largely through teacher collaboration (TC). On the other hand, when schools implement democratic cogovernance, they introduce the decentralized characteristics associated with Western individualistic culture, thus allowing teachers to increase their self-control, which may motivate teachers through teacher autonomy (TA). According to conservation of resources (COR) theory (Hobfoll, 2001), TC is viewed as an exogenous social resource. The top-down power flow associated with this situation transforms TA into an exogenous material resource. Ultimately, different types of exogenous resources flow toward endogenous resources, such as teacher self-efficacy (TSE). However, this hypothesis has not yet been tested on the basis of empirical research. Therefore, it is necessary to explore the hierarchical relationship and levels of influence among the three different motivations in terms of the influence of DL on TI within specific cultural and work environments.

This study explores the relationship between DL and TI in the Chinese context and examines the roles played by TA, TC, and TSE in this relationship. The contributions of this study are threefold. First, this research aims to determine whether the implementation of DL can promote TI in Chinese schools, specifically with the goal of clarifying the controversy concerning the relationship between these two factors on the basis of empirical research. Second, to investigate the “black box” of the process through which DL stimulates TI in the Chinese context, this study integrates SDT with COR with the aim of exploring the hierarchical relationships among TA, TC, and TSE. Third, this study provides practical path references that Chinese schools can use to stimulate TI among junior high school teachers.

## Theoretical background and hypotheses

In this section, in addition to presenting the conceptual and operationalized meanings of each variable, we describe the associations among these variables and propose relevant research hypotheses on the basis of the theoretical framework underlying this study as well as the results of previous empirical research.

### Distributed leadership and teaching innovation

#### Distributed leadership

Traditional leadership approaches have indicated that leaders should engage in “personal heroism” behavior, particularly in the case of leaders who have not only the ability to shape followers' work attitudes and behaviors but also the responsibility to lead their followers to work toward the achievement of organizational goals (Liu et al., 2021; Berraies and Zine El Abidine, 2019). However, the success of an organization does not depend solely on individual skills and characteristics. In an era that is characterized by a knowledge-based economy, collective intelligence is essential to schools' ability to achieve their developmental goals (Berraies et al., 2021). The empirical research conducted by Harris highlighted the limitations of school power by focusing on principals, thus leading Harris to claim that granting leadership to school members is the primary task of a successful principal (Harris, 2004). Specifically, school teacher teams are characterized by complexity and professionalism. It is difficult for principals to master all types of knowledge, and they cannot cope with school tasks at different stages quickly solely through their own efforts. Rather, leaders must rely on team strength and confer leadership power and functions on different members of the organization in light of their expertise in different situations, thereby distributing leadership power to the whole team with the goal of enabling the team to cope with various tasks and challenges.

Since DL represents a modern approach to leadership, no consensus has yet been reached regarding its conceptual meaning. Previous definitions of this term have been proposed from two main perspectives. One such perspective views DL as the product of interactions among school leaders, followers, and the situations in which they are embedded (Spillane et al., 2001); in contrast, the other perspective views DL as a bottom-up, highly inclusive, and participatory decision-making practice (Harris et al., 2007; Goksoy, 2016). The former perspective emphasizes the situational adaptability and dynamic evolution of DL. This perspective thus suggests that different schools must identify their unique contexts to determine the model of DL that can ensure their success most effectively. From this perspective, DL is context-specific and difficult to measure. In comparison, the latter perspective emphasizes the general characteristics of DL, namely, the secondary distribution of school autonomy to relevant subjects with the goals of achieving cogovernance at multiple levels and establishing democratic management through the participation of teachers, students, parents, and other relevant actors (OECD, 2019a). This perspective thus provides a realistic framework for efforts to explore DL in many circumstances (Liu et al., 2021). This study seeks to clarify the relationships among relevant variables, whose conceptual definitions are based on measurability. Moreover, without considering the specific context of each school, we select the second definition. In addition, most previous studies on this topic have explored DL from the perspective of principals (Liu et al., 2021; Amels et al., 2021). In contrast, this study focuses on the perspective of teachers, which is viewed as a teacher-level variable since the degree of DL depends on teachers' perceptions of the levels of power and responsibility sharing that characterize school management decisions (Gronn, 2000).

#### Teaching innovation

As a result of ongoing advancements in science and technology and the intensification of educational and teaching reforms in various countries, TI has been identified as a key area of interest for both educators and scholars. In the literature, two main definitions of TI have been proposed. The first definition identifies TI as the process of generating new ideas and transforming them into teaching behaviors and practices (Kundu and Roy, 2016; Messmann and Mulder, 2012). The second definition claims that TI refers to the use of new information technology by teachers in the process of teaching (Chou et al., 2019; Loogma et al., 2012). The former perspective emphasizes the generation and implementation of new ideas; however, it does not stipulate an excessive number of restrictions on the notion of “new.” This conception of novelty can include new teaching technologies, new teaching methods, and new teaching content, among other possibilities (Cai and Tang, 2022; O'Shea, 2021). In contrast, the latter perspective is limited to the use of new information technology in teaching practice. Since this study does not focus on teaching technology innovation, the former definition is more in line with the concept investigated in this research. Accordingly, in our study, TI is defined as teacher behavior that incorporates new teaching approaches into teaching practices and deviates from the traditional teaching mode with the goal of encouraging students to develop high-level skills (such as critical thinking, group cooperative learning, and problem-solving ability) (O'Shea, 2021; OECD, 2019b).

#### The relationship between distributed leadership and teaching innovation

In the collectivist culture that characterizes China, DL is a new leadership model that has evolved as the education system used in China has been modernized in recent years. Few studies have provided direct empirical evidence concerning the relationship between DL and TI. However, we can obtain a glimpse of this topic on the basis of the indirect evidence that has been presented.

DL emphasizes the importance of empowering teachers and sharing leadership responsibilities, which is crucial with respect to efforts to promote TI (Harris, 2007; Davison et al., 2013; Berraies et al., 2021). Empirical research that has been conducted in collectivist cultures has reported that DL not only enhances individual TI but also cultivates team innovation among teachers (Teng et al., 2024). In collectivist societies, such as those that characterize many Asian countries, individuals tend to perceive themselves as interconnected and related to others, and they strongly emphasize the importance of adhering to social norms and achieving social harmony. When leadership responsibilities are widely distributed among teachers, the values associated with obedience to leadership directives and the sense of organizational belonging that can be cultivated by effective leadership can significantly enhance teachers' job satisfaction and enthusiasm (Hulpia et al., 2009b) while simultaneously promoting their independent exploration and innovative teaching practices (O'Shea, 2021).

Accordingly, this study proposes the following hypothesis:

H1: Distributed leadership has a positive effect on TI in collectivist cultures.

### The parallel mediating roles of teacher autonomy, teacher collaboration, and teacher self-efficacy according to SDT

SDT provides a broad conceptual framework that can be used to explore how external incentives stimulate individual innovation behavior and has been recognized by many scholars (Vansteenkiste et al., 2007; Chongxin and Frenkel, 2012; Brière et al., 2021). This theory posits that innovation refers to self-initiated behavior that is inherently driven by autonomous motivation. Only external motivations that satisfy three basic psychological needs of individuals can generate such autonomous motivation, thereby fostering TI (Deci and Ryan, 2000; Amabile and Pratt, 2016). In this context, the need for autonomy pertains to an individual's perception of control and freedom in their actions; the need for competence relates to one's belief in one's ability to perform challenging tasks successfully and attain the desired outcomes; and the need for relatedness involves a sense of mutual respect for and interdependence with others (Ryan et al., 2019).

Whether in the context of individualism or collectivism, SDT provides a powerful theoretical account (Van den Broeck et al., 2016). For example, Messmann et al. (2022) investigated Dutch teachers in an individualistic culture and explored the impact of leadership styles on TI on the basis of the three basic psychological needs posited by SDT. In turn, Cai and Tang (2022) investigated Chinese teachers in a collectivist culture and explored the impact of the school climate on TI on the basis of these three basic psychological needs.

On the basis of SDT, this study develops a parallel mediation model that includes three parallel mediating variables—TA, TC, and TSE—that link DL to TI. First, DL might promote TI by enhancing TA. DL reflects the transformation of the organizational mechanism underlying school leadership from a hierarchical and centralized approach to a flat and decentralized approach, thus enabling teachers to experience autonomy in their work. Hsieh et al. (2024) investigated 2,451 teachers in Taiwan and reported that these teachers felt more personal autonomy in a supportive school environment and in the context of DL. These authors conducted a statistical analysis that verified that DL has a significant positive effect on TA. According to SDT, the more autonomy that teachers possess, the greater their corresponding autonomous motivation becomes, thus increasing their effectiveness at creative tasks such as those associated with school improvement and transformation (Buske, 2018). Lv and Zhi (2020) surveyed 318 Chinese teachers and reported that TA significantly positively impacts TI. TA encompasses the latitude that teachers have to determine course content, assign student tasks, select instructional methods, assess student learning, and manage classroom discipline (Vangrieken and Kyndt, 2020; OECD, 2019c).

Second, DL might promote TI by enhancing TC. TC depends heavily on leadership style. Liu and Werblow (2019) conducted an empirical analysis of large-scale international survey data drawn from the 2013 TALIS, and the results indicated that DL significantly promotes TC. An empirical study of Asian teachers in the context of a collectivist culture reached the same conclusion (Teng et al., 2024). According to these studies, DL ensures the extensive spread of leadership activities among different teachers, thereby encouraging them to play the role of leaders, take responsibility for corresponding leadership functions, and participate jointly in efforts to determine developmental goals for the school; ultimately, this approach leads to the establishment of a culture of TC (Harris and DeFlaminis, 2016; Harris et al., 2007). In a collaborative atmosphere, teachers exchange resources and expertise, share professional experiences, and support their peers through collaboration, thus allowing them to experience a strong sense of belonging. According to SDT, the stronger this sense of belonging is, the more actively teachers implement innovation in pursuit of the common goal of school improvement (Cai and Gong, 2019). An empirical study of 132,376 teachers from 15 countries in both the East and the West revealed that TC has a significantly positive effect on TI (Lin, 2022). TC involves the sharing of teaching-related information among colleagues and mutual learning experiences (Geijsel et al., 2009; Çoban et al., 2023; OECD, 2019c).

Third, DL might promote TI by enhancing TSE. The principal distributes decision-making and leadership power among teachers, thus representing a type of trust as well as an affirmation of teachers' ability to improve TSE effectively (Hulpia et al., 2009a; Muijs et al., 2004). On the basis of both international survey data and data obtained in Asian countries, researchers have confirmed the positive effect of DL on TSE (Sun and Xia, 2018; Teng et al., 2024). According to SDT, teachers who exhibit higher levels of self-efficacy are more proactive and enthusiastic about accepting challenging tasks, and they exhibit higher levels of commitment to teaching and engagement with the profession (Chesnut and Burley, 2015). A study on enterprise employees revealed that their self-efficacy significantly predicts their innovative behavior (Tierney and Farmer, 2002). In Asian countries and Chinese school organizations, TSE has also been proven to have a critical effect on TI (Cai and Tang, 2021; Teng et al., 2024). TSE reflects a teacher's ability to achieve effective classroom management, instruction, and student engagement (Tschannen-Moran and Hoy, 2001; Sun and Xia, 2018; OECD, 2019c; Teng et al., 2024).

Accordingly, this study proposes the following hypotheses:

H2a: Teacher autonomy mediates the relationship between DL and TI in collectivist cultures.

H2b: Teacher collaboration mediates the relationship between DL and TI in collectivist cultures.

H2c: Teacher self-efficacy mediates the relationship between DL and TI in collectivist cultures.

### The chain mediation roles of TA, TC, and TSE from the perspectives of SDT and COR in the context of contemporary Chinese culture

A parallel mediation model consisting of TA, TC, and TSE with respect to the relationship between DL and TI was constructed on the basis of SDT. In the past, researchers who have adopted a binary perspective on cultural opposition have viewed individualistic and collectivistic cultures as mutually exclusive and antagonistic. Scholars have argued that TA, as a component of individualistic culture, does not influence the implementation of TI among Chinese teachers (Cross and Markus, 1999; Cai and Tang, 2022). Collectivist cultures, which are common in Asian countries such as China, emphasize group consistency and obedience. In organizations that are characterized by high levels of power distance, subordinates tend to adhere to established rules and maintain order stability, thus leading them to respect and obey the orders of their leaders with the aims of maintaining harmonious interpersonal relationships and achieving collective goals. In contrast, individualistic cultures, which are common in Western countries, focus on personal independence and the expression of intrinsic characteristics, thus embodying a culture and set of values that emphasize self-goals, uniqueness, and self-control. The organizational structure that matches this culture is characterized by decentralized low power distance and a flat management approach (Hofstede, 2001; Oyserman et al., 2002).

However, recent studies have noted that the elements of collectivist and individualistic cultures are not mutually exclusive but rather coexist symbiotically (Vangrieken et al., 2017; Lin, 2022). This view is in line with the current development status of China. China is currently in a critical period, in which the nation focuses on increasing its educational ability; the core strategy used by the nation in this context involves promoting educational modernization, including with respect to school management. The modernization of school management does not involve a complete change with respect to the traditional bureaucratic management model but rather emphasizes the integration of bureaucracy and democratic cogovernance at the grassroots level. Posselt (2016: p. 21, 113) referred to this new model as consultative bureaucracy. The implementation of DL within the traditional bureaucratic system is representative of such consultative bureaucracy. According to this management model, general administrative decision-making continues to rely on institutional and authoritative power hierarchies, thus reflecting collectivist cultural elements, whereas decisions that pertain to professional projects such as educational and teaching reforms rely on empowered teachers, thereby reflecting individualist cultural elements. It is thus reasonable to infer that DL initially activates TC and TA. Furthermore, TC, which takes place through discussions of teaching issues and efforts to share practical knowledge, enhances educators' confidence in their teaching practices and the overall quality of classroom instruction (Geijsel et al., 2009; Çoban et al., 2023). Additionally, teachers who have attained a high degree of classroom autonomy are more likely to be motivated to refine their teaching practices and pursue professional development, thus ultimately increasing their self-efficacy (Wermke et al., 2019; Choi and Mao, 2021). Therefore, at the current step in the process of educational modernization in China, the relationships among TC, TA, and TSE may not be parallel but rather hierarchical.

COR theory views TA, TC, and TSE as different types of resources, thereby providing an explanatory framework for the flow of resources that occurs in the process of implementing DL with the goal of stimulating TI among teachers in the Chinese context. The fundamental tenet of COR theory posits that all human beings inherently possess the propensity to acquire, protect, and cultivate resources that are of paramount significance with respect to their behavioral intentions (Hobfoll et al., 2018). During the deep phase of educational reform, the implementation of TI often consumes a significant amount of teachers' resources as a result of the risks and uncertainties that are inherent in the innovation process, thus leading to exhaustion. In this context, the implementation of DL can, on the one hand, grant teachers more autonomy and enhance their sense of control over decision-making and development within the school. This TA, which is the result of the flow of power between superiors and subordinates, enables teachers to accumulate material resources and activates individualistic cultural elements. On the other hand, as a result of their respect for and obedience to leadership, teachers tend to perceive DL as an order from higher authorities or a collective goal. To achieve this collective goal more effectively, teachers collaborate and share teaching and work experiences within the team with the aim of obtaining more social resources, thereby activating collectivist cultural elements. According to COR theory, both material resources and social resources are exogenous resources that exist independently of individuals in the context of social interactions (Hobfoll, 2001). On the basis of the characteristics of resource flow, exogenous resources can enhance endogenous resources such as TSE, thereby promoting an increase in the value of teachers' own resources (Hobfoll, 2002). Teachers can transform these resources into high-performance TI behaviors, thus enabling them to earn high recognition from their leaders; in turn, such recognition fosters a virtuous cycle of resource acquisition, protection, and construction. Accordingly, this study aims to develop a chain mediation model in which TA and TC concurrently impact TSE within the framework of the influence of DL on TI.

Accordingly, this study proposes the following hypotheses:

H3a: The influence of teacher autonomy on teacher self-efficacy has a chain mediating effect on the relationship between DL and TI in collectivist cultures.

H3b: The influence of teacher collaboration on teacher self-efficacy has a chain mediating effect on the relationship between DL and TI in collectivist cultures.

## Methods

### Data sources

This study involved a secondary data analysis that was conducted by reference to the Teaching and Learning International Survey (TALIS) 2018 database (SPSS_2018_international), which is collected by the Organization for Economic Cooperation and Development (OECD, 2018). Our reason for choosing TALIS2018 is that this database aims to provide researchers with international data concerning school leadership as well as teachers' attitudes practices and behaviors (OECD, 2019d); furthermore, it encompasses measurements of key components in this study. In addition, the data collection process for TALIS2018 employed probability proportionate to size sampling. Scholars have demonstrated that data collected on the basis of this sampling technique exhibit strong representativeness, mitigate sampling errors, and guarantee the quality of associated research (Liu et al., 2021).

TALIS2018 collected data concerning teachers from 48 participating countries and economies, including teachers from Shanghai, China. The focus of this research on Shanghai is justified by the status of this municipality as a leader in the field of educational reform in China. As the nation's economic and educational center, Shanghai has implemented numerous innovative educational policies, thereby attracting significant attention at both the domestic and international levels. Its modern schools and widespread use of DL models identify it as an exemplary case for this effort to examine the modernization of school management within a collectivist cultural context. Moreover, Shanghai's outstanding performance on international assessments, particularly the Program for International Student Assessment (PISA), in which context students from this municipality consistently excel in the fields of mathematics, science, and reading, highlights its relevance as a representative sample for this research on educational quality and practices in China.

This study used data drawn from the “core” TALIS collection to investigate teachers at the lower secondary level. A total of 3,976 teachers in Shanghai, China, constituted the sample investigated in this study, which included 2,941 females (74%) and 1,035 males (26%). With respect to the age of the teachers included in this sample, 16.4% were under the age of 30 years, 33.1% were between the ages of 30 and 39 years, 35.7% were between the ages of 40 and 49 years, 14.2% were between the ages of 50 and 59 years, and 0.6% were 60 years old or older. With respect to teaching experience, 12.3% of these teachers had <5 years of experience, 31.3% had between 6 and 15 years, and 51.8% had more than 15 years; the remaining 4.6% of teachers exhibited missing data in this regard. The levels of education attained by the teachers included in the sample were as follows: 0.9% of these teachers had obtained associate degrees, whereas 98.8% had obtained bachelor's degrees or higher (missing data accounted for 0.3% of the sample in this context).

### Measures

The purpose of this study is to investigate the relationship between DL and TI, as well as the mediating roles played by TA, TC, and TSE in this context. Consequently, this study focuses on five variables drawn from the TALIS2018 teacher dataset: the dependent variable TI; the mediating variables TA, TC, and TSE; and the independent variable DL. The survey items employed for the latent variables collected by the OECD are detailed in Appendix A, and the reliability and validity of the scales are presented below.

#### Dependent variable

The outcome variable used in this study is TI. The teacher survey includes six questions that are used to assess the frequency of teachers' engagement in two subscales: cognitive activation (TT3G42E, TT3G42F, TT3G42G, and TT3G42H), which refers to teaching practices that foster critical thinking and problem-solving skills among students, and advanced activities (TT3G42O and TT3G42P), which involve collaborative student work and the integration of technology into projects or assignments, which typically demand more time from both teachers and students than do traditional instructional methods (O'Shea, 2021). All the items are scored on a four-point Likert scale, in which a score of 1 indicates “Never or almost never,” a score of 2 indicates “Occasionally,” a score of 3 indicates “Frequently,” and a score of 4 indicates “Always.” The use of such items to measure TI has been validated on the basis of psychometric testing (Pan et al., 2024). A reliability analysis of this scale revealed that Cronbach's alpha coefficient was 0.808. Furthermore, a confirmatory factor analysis (CFA) indicated that the scale exhibited good construct validity (χ^2^/df = 15.98, GFI = 0.991, CFI = 0.987, TLI = 0.972, RMSEA = 0.061, SRMR = 0.022).

#### Mediating variables

The mediating variables included in this study are TA, TC, and TSE. The TA survey includes five items that ask teachers how strongly they agree or disagree with claims concerning their control over various factors (TT3G40A, TT3G40B, TT3G40C, TT3G40D, and TT3G40E) (OECD, 2019c: p. 302). All these items are scored on a four-point Likert scale, in which a score of 1 indicates “Strongly disagree,” a score of 2 indicates “Disagree,” a score of 3 indicates “Agree,” and a score of 4 indicates “Strongly agree.” The technical report provided by the OECD indicates that the internal consistency reliability of the scale for most countries is above or close to 0.7, and the CFA model fit indices for the scale in most countries are acceptable (OECD, 2019c: p. 308–311). As part of this study, the reliability and validity of the scale were tested; the results revealed a Cronbach's alpha coefficient of 0.922, and the CFA indicated a good model fit (χ^2^/df = 5.119, GFI = 0.998, CFI = 0.999, TLI = 0.997, RMSEA = 0.032, SRMR = 0.005).

The TC scale features four items that indicate how often, on average, teachers engage in TT3G33D, TT3G33E, TT3G33F, and TT3G33G (OECD, 2019c: p. 252). All the items are scored on a six-point Likert scale, in which a score of 1 indicates “Never,” a score of 2 indicates “Once per year or less,” a score of 3 indicates “2–4 times per year,” a score of 4 indicates “5–10 times per year,” a score of 5 indicates “1–3 times per month,” and a score of 6 indicates “Once per week or more.” The technical report provided by the OECD indicates that the internal consistency reliability of the scale for most countries is above 0.7, and the CFA model fit indices for the scale in most countries are acceptable (OECD, 2019c: p. 252–256). As part of this study, the reliability and validity of the scale were tested; the results revealed a Cronbach's alpha coefficient of 0.814, and the CFA indicated a good model fit (χ^2^/df = 8.556, GFI = 0.998, CFI = 0.997, TLI = 0.992, RMSEA = 0.044, SRMR = 0.011).

The TSE variable was measured in terms of three subscales: self-efficacy in classroom management (SECLS, which includes TT3G34D, TT3G34F, TT3G34H, and TT3G34I), instruction (SEINS, which includes TT3G34C, TT3G34J, TT3G34K, and TT3G34L), and student engagement (SEENG, which includes TT3G34A, TT3G34B, TT3G34E, and TT3G34G) (OECD, 2019c: p. 285). All the items are scored on a four-point Likert scale in which a score of 1 indicates “Not at all,” a score of 2 indicates “To some extent,” a score of 3 indicates “Quite a bit,” and a score of 4 indicates “A lot.” The technical report provide by the OECD indicates that the internal consistency reliability of the scale for most countries is above or close to 0.7, and the CFA model fit indices for the scale in most countries are acceptable (OECD, 2019c: p. 285–293). As part of this study, the reliability and validity of the scale were tested; the results revealed a Cronbach's alpha coefficient of 0.955, and the CFA indicated a good model fit (χ^2^/df = 21.564, GFI = 0.959, CFI = 0.978, TLI = 0.968, RMSEA = 0.072, SRMR = 0.025).

#### Independent variable

DL is included in this study as an independent variable. This scale used to measure this factor includes five items that ask respondents how strongly the teacher agrees or disagrees with TT3G48A, TT3G48B, TT3G48C, TT3G48D, and TT3G48E. All these items are scored on a four-point Likert scale in which a score of 1 indicates “Strongly disagree,” a score of 2 indicates “Disagree,” a score of 3 indicates “Agree,” and a score of 4 indicates “Strongly agree.” The use of such items to measure DL has been emphasized by the OECD (2019a,c) and other scholars (Sun and Xia, 2018; Wu, 2021; Teng et al., 2024). A reliability analysis of this scale revealed that the Cronbach's alpha coefficient was 0.941. Furthermore, a CFA demonstrated that the scale exhibited good construct validity (χ^2^/df = 12.671, GFI = 0.995, CFI = 0.998, TLI = 0.994, RMSEA = 0.054, SRMR = 0.006).

#### Control variables

The control variables referenced in this study include teacher gender, years of teaching experience, educational level, school type (public or private), school location (urban or rural), whether teaching was the respondent's first career choice, and respondents' perceptions of the effectiveness of professional development programs. Previous research has reported that both teachers' demographic characteristics and their participation in professional learning communities (Liu et al., 2022; Teng et al., 2024) significantly influence their TI.

### Analytical approach

As part of this study, SPSS 27 software was initially used for data cleaning, descriptive statistics, reliability and validity testing, an analysis of common method variance (CMV), and a regression analysis. Structural equation models were subsequently constructed with the assistance of AMOS 24.0 software to explore the relationships of DL, TA, TC, and TSE with TI in further detail and to compare the fit indices of the chain mediation model with those of the parallel mediation model. Finally, the bootstrapping technique was used to test the independent mediating effects of TA, TC, and TSE, as well as the chain mediating effects of TA → TSE and TC → TSE.

## Results

### Descriptive statistics

The descriptive statistics are presented in Table 1. The results of scored on a four-point scale and reveal that the level of TA is the highest (M = 3.39), followed by TSE (M = 3.312) and DL (M = 3.024). In contrast, the comparatively lower level of TI observed in this context (M = 2.466) highlights the necessity of exploring the mechanism used to promote TI during the challenging phase of educational and teaching reform in China. On a six-point scale, the average score for TC is 4.003, thus indicating a moderate level of collaboration among teachers.

**Table 1:** Means, standard deviations, and correlations.

**Variables**	**M**	**SD**	**1**	**2**	**3**	**4**	**5**
1. Distributed leadership	3.024	0.598	1				
2. Teacher collaboration	4.003	1.219	0.304^**^	1			
3. Teacher self-efficacy	3.312	0.539	0.295^**^	0.272^**^	1		
4. Teacher autonomy	3.390	0.459	0.244^**^	0.193^**^	0.343^**^	1	
5. Teaching innovation	2.466	0.539	0.204^**^	0.163^**^	0.283^**^	0.141^**^	1

** *P* < 0.01.

In addition, the correlation coefficients among the variables range from 0.141 to 0.343 and are significant at the 0.01 level, thus indicating significant positive correlations among all the variables; furthermore, all the correlation coefficients are below the threshold of 0.5 used to determine multicollinearity. Moreover, this study tests for multicollinearity by conducting a variance inflation factor (VIF) analysis of the regression model. The results indicate that the VIF values pertaining to DL, TC, TA, and TSE are 1.186, 1.154, 1.17, and 1.234, respectively. All these values are < 3, thus indicating a low likelihood of multicollinearity issues in this study.

### Measurement model

#### Reliability and validity tests of the constructs

Table 2 indicates that the composite reliability (CR) values for all the constructs range from 0.866 to 0.961, thus exceeding the acceptable threshold of 0.70 recommended by Hair et al. (2011). Additionally, the average variance extracted (AVE) values range from 0.519 to 0.811, thus exceeding the 0.50 threshold proposed by Hair et al. (2011). These results provide evidence to support the internal consistency and convergent validity of the constructs.

**Table 2:** Reliability and validity of the main constructs.

**Variables**	**CR**	**AVE**	**1**	**2**	**3**	**4**	**5**
1. Distributed leadership	0.955	0.811	0.901				
2. Teacher collaboration	0.878	0.644	0.304	0.802			
3. Teacher self-efficacy	0.961	0.675	0.295	0.272	0.822		
4. Teacher autonomy	0.944	0.771	0.244	0.193	0.343	0.878	
5. Teaching innovation	0.866	0.519	0.204	0.163	0.283	0.141	0.720

To evaluate discriminant validity, we used the method developed by Fornell and Larcker (1981). As indicated in Table 2, the square root of the AVE for each construct (i.e., the diagonal values) is greater than the corresponding correlation coefficients with all other constructs (i.e., the off-diagonal values), thus confirming robust discriminant validity among the constructs.

#### Analysis of common method variance

Two methods were used to examine CMV in this study. The first method involved the use of Herman's single-factor CFA test to examine CMV, namely, by performing a single-factor CFA with respect to all the items (Podsakoff et al., 2003). If the single-factor model exhibits a good fit, this finding indicates serious CMV among the variables; otherwise, no such CMV is indicated. Table 3 reveals that the fit index of the single-factor model did not reach an acceptable level. The second test method used in this context was the factor control method, which allows each measurement item to load not only onto its own theoretical factor but also onto a latent factor (Podsakoff et al., 2003), thus leading to the emergence of a new model labeled as Ma. If the new model (Ma) is significantly better than the theoretical model (i.e., the 5-factor model), severe CMV is indicated among the variables; otherwise, no such CMV is indicated. Table 3 reveals that, in comparison with those associated with the 5-factor model, the goodness-of-fit index (GFI), comparative fit index (CFI), and Tucker–Lewis index (TLI) associated with Ma increased by 0.036, 0.026, and 0.028, respectively, which were much lower than the threshold of 0.05 (Little, 1997). The test results obtained through the use of these two methods indicate the absence of serious CMV among the variables included in this study.

**Table 3:** Results of the test of common method variance.

**Model**	**χ^2^/df**	**GFI**	**CFI**	**TLI**	**RMSEA**	**SRMR**
5-factor (DL;TC;TSE;TA;TI)	17.079	0.936	0.957	0.948	0.064	0.028
4-factor (DL+TI;TC;TSE;TA)	25.336	0.907	0.933	0.921	0.078	0.056
3-factor (DL;TC+TSE+TA;TI)	107.477	0.672	0.7	0.656	0.164	0.142
2-factor (DL+TI;TC+TSE+TA)	112.912	0.661	0.681	0.639	0.168	0.149
1-factor (DL+TI+TC+TSE+TA)	207.462	0.467	0.407	0.333	0.228	0.202
M_a_	8.4	0.972	0.983	0.976	0.043	0.020

DL, Distributed leadership; TA, Teacher autonomy; TC, Teacher collaboration; TSE, Teacher self-efficacy; TI, Teaching innovation.

The TALIS 2018 questionnaire was designed to mitigate the CMV resulting from both social desirability bias and common rater effects through the use of various means, such as reverse-coded items, efforts to ensure participant anonymity, and an emphasis on the fact that no answers were right or wrong. The results of this analysis confirmed that the CMV in this study remained within acceptable limits. Importantly, however, since the questionnaire data rely exclusively on self-reports provided by teachers, the potential risk of CMV cannot be eliminated entirely.

### Hypothetical model

#### The relationship between distributed leadership and teaching innovation

A stepwise regression on the basis of the ordinary least squares method was performed to facilitate an initial examination of the relationship between DL and TI while controlling for teacher demographics and school characteristics. This analysis also aimed to explore the possible mediating roles played by TA, TC, and TSE in this relationship. The results are presented in Table 4.

**Table 4:** Stepwise regression of the effect of distributed leadership on teaching innovation.

**Independent variable**	**TI (model 1)**	**TA (model 2)**	**TC (model 3)**	**TSE (model 4)**	**TSE (model 5)**	**TI (model 6)**
Female	−0.012 (0.021)	0.049^**^ (0.018)	0.052 (0.035)	0.023 (0.020)	0.002 (0.018)	−0.021 (0.020)
Years of teaching experience	0.004^***^ (0.001)	−0.005^***^ (0.001)	−0.004^*^ (0.002)	0.008^***^ (0.001)	0.010^***^ (0.001)	0.003^**^ (0.001)
Post-graduate	0.020 (0.027)	−0.040 (0.023)	0.025 (0.045)	−0.007 (0.025)	0.002 (0.023)	0.022 (0.026)
Teaching as first career choice	0.023 (0.028)	−0.022 (0.024)	0.088 (0.048)	0.030 (0.027)	0.027 (0.025)	0.014 (0.028)
Effective professional development	−0.321^***^ (0.054)	−0.149^***^ (0.046)	−0.670^***^ (0.091)	−0.295^***^ (0.051)	−0.173^***^ (0.048)	−0.224^***^ (0.053)
Public school	−0.106^***^ (0.026)	−0.011 (0.022)	0.053 (0.043)	−0.062^*^ (0.024)	−0.064^**^ (0.023)	−0.095^***^ (0.025)
Urban school	0.012 (0.008)	0.019^**^ (0.007)	0.040^**^ (0.014)	0.015 (0.008)	0.004 (0.007)	0.006 (0.008)
DL	0.190^***^ (0.016)	0.201^***^ (0.014)	0.464^***^ (0.027)	0.271^***^ (0.015)	0.156^***^ (0.015)	0.105^***^ (0.017)
TA					0.312^***^ (0.018)	0.037 (0.021)
TC					0.113^***^ (0.009)	0.043^***^ (0.010)
TSE						0.210^***^ (0.019)
Constant	2.095^***^ (0.169)	3.152^***^ (0.145)	2.801^***^ (0.284)	2.674^***^ (0.161)	1.375^***^ (0.161)	1.295^***^ (0.179)
*N*	3, 363	3, 363	3, 363	3, 363	3, 363	3, 363
*R* ^2^	0.072	0.084	0.123	0.132	0.246	0.125
Adjusted *R*^2^	0.069	0.082	0.121	0.130	0.244	0.122
*F*	32.377	38.659	58.617	63.857	109.652	43.377

DL, Distributed leadership; TA, Teacher autonomy; TC, Teacher collaboration; TSE, Teacher self-efficacy; TI, Teaching innovation; the reference groups for the variables female, public schools, and urban schools were male, private schools, and rural schools, respectively;

* p < 0.05,

** p < 0.01,

*** p < 0.001; standard errors are shown in parentheses.

Model 1 assesses the effect of DL on TI, taking into account only the control variables, in which context TI is used as the dependent variable. Models 2 and 3 and Models 4 and 5 focus on the influence of DL on the three mediating variables (TA, TC, and TSE), in which context each of these three variables are treated as the dependent variable in turn. Model 6 represents the final full model, which analyzes the influence of DL on TI when both the mediating and control variables are included.

According to Model 1, after teacher demographics and school characteristics are controlled, DL continues to have a significant positive effect on TI (β = 0.19, *p* < 0.001). This finding suggests that a higher level of DL in schools is associated with increased stimulation of TI among teachers.

Table 4 indicates that DL has significant positive effects on TA, TC, and TSE. While TC and TSE significantly influence TI, TA (β = 0.037, *p* > 0.05) does not have a significant effect, thus indicating that TC and TSE (but not TA) may play mediating roles in this context. Furthermore, TA and TC are positively correlated with TSE, and the full model indicates that the regression coefficients of DL on TI remain significant even after the inclusion of TA, TC, and TSE as mediating variables. These finding suggest the possible existence of hypothesized chain mediation effects in this context, according to which TA may not impact TI directly but instead influence that factor indirectly via TSE. To validate these mediating effects, a structural equation model was used.

#### Test of the parallel mediating effects on the basis of structural equation models

To compensate for the limitations of stepwise regression in the context of mediation testing and to enhance the ability of this research to control for measurement errors, this study employs a structural equation model that is rooted in the bootstrap method.

**Figure 1:**
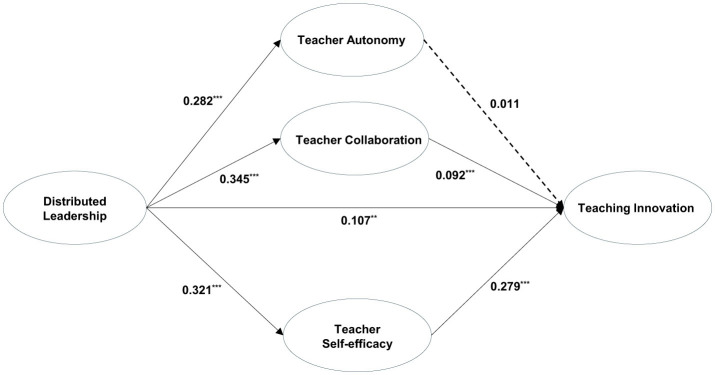
Standardized SEM results concerning the parallel mediating model. The dashed lines indicate non-significant paths, **P < 0.01, ***P < 0.001.

This study draws on the framework of SDT and proposes three parallel mediating variables—TA, TC, and TSE—with respect to the relationship between DL and TI, thus developing a parallel mediation model. The results indicate that, with the exception of the standardized root mean square residual (SRMR) (0.087 > 0.08), all the fit indicators of the model fall within an acceptable range (χ^2^ = 3053.938, df = 145, χ^2^/df = 21.062, GFI = 0.92, CFI = 0.945, TLI = 0.935, RMSEA = 0.071). On the basis of 5,000 bootstrap resamples, 95% confidence intervals are calculated. The results are presented in Table 5, revealing that the direct effect of DL on TI is significant; in addition, the mediating effects of TC and TSE in this context are significant. However, the results do not indicate a significant mediating effect of TA (mediating effect TA = 0.003, *P* > 0.05) since TA does not significantly predict TI (β_TA − TI_ = 0.011, *p* > 0.05). These results support H2b and H2c but do not support H2a. The path diagrams are illustrated in Figure 1.

**Table 5:** Bootstrapping results regarding the parallel mediation model.

**Path**	**Estimate**	**SE**	**Bootstrapping** **Percentile 95% CI**		***P***
			**Lower**	**Upper**	
**Standard direct effect**					
DL → TI	0.107	0.022	0.064	0.149	0.001
DL → TA	0.282	0.017	0.247	0.315	0.000
DL → TC	0.345	0.018	0.31	0.381	0.000
DL → TSE	0.321	0.018	0.286	0.355	0.000
TA → TI	0.011	0.02	−0.029	0.05	0.604
TC → TI	0.092	0.021	0.053	0.13	0.000
TSE → TI	0.279	0.02	0.24	0.322	0.000
**Standard indirect effect**					
DL → TA → TI	0.003	0.006	−0.009	0.14	0.604
DL → TC → TI	0.032	0.007	0.015	0.045	0.000
DL → TSE → TI	0.09	0.008	0.075	0.108	0.000

DL, Distributed leadership; TA, Teacher autonomy; TC, Teacher collaboration; TSE, Teacher self-efficacy; TI, Teaching innovation.

The results of the study mentioned above indicate that TC and TSE play significant mediating roles in the relationship between DL and TI, whereas the mediating role played by TA in this context is not significant. This phenomenon may be the result of the emphasis on group integration that characterizes collectivist cultures, in which individuals prioritize collective goals. In this cultural context, individual autonomy often conflicts with collective goals and norms, thus causing teachers to be reluctant to take risks in terms of altering traditional teaching practices. When TA is decoupled from collective norms, their innovative potential may be suppressed, resulting in a non-significant direct impact on TI in this context. Furthermore, the parallel mediation model constructed on the basis of SDT reveals that the model fit index SRMR exceeds the acceptable range, thus indicating a low level of fit between the model and the data; in turn, this finding may suggest an unreasonable model structure. Therefore, this parallel mediation model requires improvement.

#### Test of the chain mediating effects on the basis of structural equation models

This study further revises the previously mentioned parallel mediation model through the integration of SDT and COR and constructs a chain mediation model that encompasses the paths TA → TSE and TC → TSE in the context of DL and TI. The results indicate that all fit indices of the chain mediation model fall within an acceptable range (χ^2^ = 2510.727, df = 143, χ^2^/df = 17.558, GFI = 0.934, CFI = 0.955, TLI = 0.947, RMSEA = 0.065, SRMR = 0.043) and surpass those associated with the parallel mediation model. Specifically, the RMSEA and SRMR values are smaller (RMSEA = 0.065 < 0.071, SRMR = 0.043 < 0.087), whereas the GFI, CFI, and TLI values are larger (GFI = 0.934 > 0.92, CFI = 0.955 > 0.945, TLI = 0.947 > 0.935). Furthermore, a significant difference was observed between these two models (Δχ^2^ = 543.211, Δdf = 2, *P* < 0.001).

**Figure 2:**
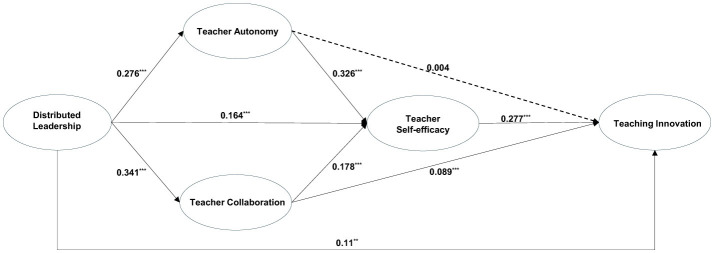
Standardized SEM results regarding the chain mediating model. The dashed lines indicate non-significant paths, **P < 0.01, ***P < 0.001.

The bootstrapping approach was used to test the chain mediating effects of TA → TSE and TC → TSE. The 95% confidence intervals were calculated on the basis of 5,000 bootstrap resamples. The results are presented in Table 6, which indicates that DL promotes TI among teachers via one direct path and four indirect paths: direct path, DL → TI; indirect path 2, DL → TC → TI; indirect path 3, DL → TSE → TI; indirect path 4, DL → TA → TSE → TI; and indirect path 5, DL → TC → TSE → TI. The value of the direct effect of DL on TI was 0.11 (*p* < 0.01), and the 95% confidence interval (CI) for the indirect paths listed above did not contain zero. Therefore, H1, H3a, and H3b were supported. The path diagrams are illustrated in Figure 2.

**Table 6:** Bootstrapping results regarding the chain mediation model.

**Path**		**Estimate**	**SE**	**Bootstrapping percentile 95% CI**		***P***	**Proportion of the effect**
				**Lower**	**Upper**		
Total effect		0.228	0.021	0.187	0.269	0.000	100%
Direct effect		0.11	0.022	0.067	0.152	0.001	48.246%
Total indirect effect		0.118	0.01	0.1	0.139	0.000	51.754%
**Standard direct effect**							
DL → TI		0.11	0.022	0.067	0.152	0.001	
DL → TA		0.276	0.017	0.241	0.309	0.000	
DL → TC		0.341	0.018	0.306	0.377	0.000	
DL → TSE		0.164	0.019	0.126	0.201	0.000	
TA → TI		0.004	0.02	−0.037	0.043	0.844	
TC → TI		0.089	0.02	0.049	0.126	0.000	
TSE → TI		0.277	0.022	0.236	0.32	0.000	
TA → TSE		0.326	0.017	0.291	0.359	0.000	
TC → TSE		0.178	0.018	0.142	0.213	0.000	
**Standard indirect effect**							
Path 1	DL → TA → TI	0.001	0.006	−0.01	0.012	0.844	0.439%
Path 2	DL → TC → TI	0.03	0.007	0.017	0.044	0.000	13.158%
Path 3	DL → TSE → TI	0.045	0.006	0.034	0.059	0.000	19.737%
Path 4	DL → TA → TSE → TI	0.025	0.003	0.02	0.031	0.000	10.964%
Path 5	DL → TC → TSE → TI	0.017	0.002	0.013	0.022	0.000	7.456%
	Path 2–path 3	−0.015	0.01	−0.035	0.004	0.113	
	Path 2–path 4	0.005	0.008	−0.009	0.02	0.509	
	Path 2–path 5	0.013	0.007	−0.001	0.027	0.073	
	Path 3–path 4	0.02	0.006	0.009	0.033	0.001	
	Path 3–path 5	0.029	0.007	0.017	0.043	0.000	
	Path 4–path 5	0.008	0.003	0.002	0.014	0.005	

DL, Distributed leadership; TA, Teacher autonomy; TC, Teacher collaboration; TSE, Teacher self-efficacy; TI, Teaching innovation.

As shown in Table 6, DL has a stronger influence on TI among teachers via the mediating path, accounting for 51.754% of the total effect, whereas the direct effect accounts for a smaller proportion of 48.246%. Significant differences exist among indirect paths 3, 4, and 5. In this context, the mediating effect of DL → TSE → TI is the strongest, at 19.737%, followed by DL → TA → TSE → TI at 10.964%, whereas the chain mediating effect of DL → TC → TSE → TI is the weakest, at 7.456%.

These results indicate that the chain mediation model outperforms the parallel mediation model and thus verify the significant mediating roles played by TA, TC, and TSE in the relationship between DL and TI. Specifically, the direct impact of TA on TI is not significant, but it indirectly influences TI via TSE. This finding indicates that in a collectivist cultural context, TA is not unimportant with regard to TI; rather, TA must meet specific prerequisites to impact TI. That is, only when TA enhances their confidence in teaching and when they believe that the implementation of TI on the basis of their own abilities can facilitate the achievement of collective goals is their TI stimulated.

## Discussion

This study investigates teachers living in Shanghai, China, which is characterized by a collectivist culture, and specifically explores whether and how DL promotes TI among teachers. The findings of this research indicate that, first, the implementation of DL in China, which is characterized by a high level of power distance, can stimulate TI. Second, to explain the mechanism by which DL affects TI, in this study, a parallel mediation model is constructed on the basis of SDT, as is a chain mediation model that combines SDT with COR. A comparison of the models reveals that the chain mediation model exhibits superior fit indices, thus indicating its superiority. Finally, the test results regarding the mediating effects associated with the chain mediation model reveal that DL stimulates TI in teachers via two independent mediation paths, i.e., TC and TSE, as well as two chain mediation paths, i.e., TA-TSE and TC-TSE. Notably, the direct effect of TA on TI is non-significant, and this factor can influence TI only via TSE.

### Theoretical implications

First, this study enriches the microfoundations of TI at the individual level. Early research on TI focused mainly on its intrinsic determinants and viewed this factor as an inherent personal quality that is evident in certain types of preference, intelligence, or personality. However, as the fields of behaviorism and social psychology have continued to advance, scholars have increasingly directed their attention to the external environments that stimulate innovative behavior (Hennessey et al., 2020). Despite the consistent findings in the literature that have indicated that leadership style represents one of the most significant facilitators of TI (Zhu et al., 2019; Teng et al., 2024; Wang and Bai, 2025), our understanding of the underlying mechanisms remains insufficient. Moreover, the role played by DL in schools, particularly in collectivist cultural contexts that are characterized by high levels of power distance, has been neglected by most studies. Therefore, this study focuses on teachers in Shanghai, which is characterized by a collectivist culture, uses SDT and COR as theoretical frameworks, and employs TA, TC, and TSE as process mechanisms with the aim of exploring the influence of DL on TI, thereby complementing the micropsychological foundations of TI.

Second, this study clarifies the relationship between DL and TI in the context of a collectivist culture, thereby addressing the controversies that have characterized previous research on this topic. Research on the relationship between DL and TI in collectivist cultures remains in its early stages. Although this topic has gradually received attention from scholars at both the domestic and international levels in recent years, most studies on this topic have remained at the stage of theoretical speculation, and relatively few empirical studies have been conducted in this context. The existing theoretical conception of this topic is characterized by conflicting perspectives that urgently require validation. The negative perspective suggests that DL contradicts the form of bureaucracy that is prevalent in collectivist cultures, which is characterized by a high level of power distance, thereby potentially increasing teachers' work burden and role conflicts; in turn, this impact can undermine TI behavior (Harris, 2003; Gong, 2023). However, the positive perspective holds that DL addresses the limitations that result from hierarchical solidification and a lack of flexibility in such a bureaucracy, including in terms of poor information transmission, slow decision-making, and low teacher participation. It can thus promote participation in decision-making by teachers and the implementation of autonomous behavior through the delegation of leadership (Teng et al., 2024; Unsworth et al., 2005). On the basis of a survey of teachers in Shanghai, this study provides empirical evidence to support the positive perspective on DL in terms of its ability to promote TI in collectivist cultures. It thus responds to the controversies that have been reported in the literature and improves our understanding of the relationship between DL and TI among teachers in collectivist cultures.

Third, this study enriches and deepens the research context and framework of SDT. SDT views autonomy, relatedness, and competence as crucial process elements, thereby offering a theoretical explanation for how external factors influence autonomous behaviors such as those associated with TI. However, the question of whether the relationships among these three process elements are parallel or hierarchical remains unanswered. Therefore, this study constructed a parallel mediation model on the basis of SDT and revised the SDT framework by reference to the characteristics of the working environments in which DL is implemented, particularly in contexts that are characterized by high levels of power distance and the resource flow characteristics posited by COR, thereby constructing a chain mediation model. A test of the model fit revealed that the level of fit between the parallel mediation model and the data was relatively low (SRMR = 0.087 > 0.08), thus indicating a need for further modification in this context. In contrast, the fit indices of the chain mediation model were all within an acceptable range and superior to those associated with the parallel mediation model. This study thus verifies that the chain mediation model constructed on the basis of SDT and COR can explain the mechanism by which DL influences TI in collectivist cultures more accurately, thereby enriching and deepening the application of SDT in such cultures.

Finally, this study examines the black box that represents the relationship between DL and TI in collectivist cultures. Previous studies have failed to provide a clear understanding of the process mechanism through which DL influences TI in collectivist cultures. This study reveals that the implementation of DL within a management structure of bureaucracy can simultaneously activate TC, which is linked with collectivist cultures, and TA, which is connected to individualist cultures. Once such TC is activated, it can not only influence TI directly but also indirectly via TSE. This finding is in line with related research (Cai and Tang, 2021; Teng et al., 2024). In contrast, the activated TA does not directly influence TI; instead, it affects TI only indirectly via TSE. This finding challenges the perspective that has been presented in previous studies, which has suggested that TI in collectivist cultures does not necessitate autonomous motivation (Cai and Tang, 2022; Cai and Gong, 2019; Church et al., 2013). In collectivist cultures, organizational goals are consistently identified as superior to individual goals. When DL confers autonomy and a sense of control over their teaching on teachers, if these teachers lack the professional competence and confidence they need to pursue organizational goals more effectively through the implementation of TI, they may feel uneasy because their innovative actions deviate from collective objectives. In these circumstances, even if teachers have received autonomy, they might adopt a mindset in which they prefer not to innovate rather than hold back the group and thus adhere to school regulations and align their teaching practices with those of their peers to the greatest extent possible. Therefore, TA does not impact TI directly unless teachers, after they receive autonomy, possess the ability and confidence to promote the realization of organizational goals by taking innovative actions, in which case this factor has a positive impact on TI. The findings of this study thus provide an important theoretical reference for efforts to take full advantage of the structural role of DL in collectivist cultures.

### Practical implications

The results of this study rely primarily on data collected from teachers in Shanghai, which is viewed as a crucial “pilot city” with respect to educational reform in China. This context allows this research to reflect, to some extent, the development trends observed in the process of educational modernization in this country. During the period when the construction of a strong educational country was comprehensively promoted in China, modernization and reform of school management were imperative. Thus, to a certain extent, the results of this study can be used as a practical reference for efforts to improve school management and stimulate TI in other regions.

First, the principal should transform the traditional hierarchical management model and actively promote the implementation of DL. On the one hand, the principal must focus on the task of enhancing their own leadership skills through self-education and professional training, thus enabling them to master the skills that are required for the implementation of DL and to develop the ability to delegate authority accurately in different organizational contexts, thereby ensuring the effective achievement of organizational goals. On the other hand, the implementation of DL involves the delegation of leadership authority to teachers, which, in turn, requires active cooperation on the part of the empowered teachers. Therefore, the cultivation of teacher leadership should not be overlooked. By enhancing the leadership capabilities of both the principal and the teachers, the comprehensive development of DL in schools can be promoted, thus increasing the efficiency of organizational management and TI.

Second, the principal should lead teachers in the process of creating a collaborative, sharing, and positive environment for interpersonal interaction. In terms of teachers' awareness of collaboration, the principal should help teachers recognize the importance of collaboration with respect to efforts to achieve organizational goals, overcome limits on individuals' professional growth, expand teaching resources, and promote a positive inclination toward collaboration among teachers. To implement collaboration, the principal should establish a collaborative resource platform that provides leadership support, peer support, and expert support for the implementation of TC.

Third, the principal should emphasize the important ability of TA to promote the process of TI and ensure that teachers are able to acquire autonomy through institutional development. While DL decentralizes power to the level of teachers, it may also have negative effects, such as the emergence of role conflicts and imbalances for teachers. To mitigate these adverse effects, the principal should create an environment that encourages teachers to make independent decisions and actively promote the establishment of governance systems that can facilitate DL. These goals can be achieved through the formulation of clear regulations and guidelines that provide psychological and behavioral support for empowered teachers.

Finally, the principal should prioritize the cultivation of TSE. The aforementioned measures at the organizational level can, to some extent, enhance teachers' professional development and self-efficacy through the establishment of TC platforms and the implementation of institutional safeguards for TA. In addition, the principal can share scientific intervention strategies with teachers to help them develop the ability to construct their own self-efficacy positively. For example, encouraging teachers to keep a journal to record events that elicit a sense of achievement in their teaching work can help them continually receive positive affirmations and motivation from within. This psychological awakening can help enhance TSE, thereby promoting TI.

### Limitations and directions for future research

This study presents important findings and has significant implications; however, it also has several limitations. First, this study analyzes the relationships among DL, TA, TC, TSE, and TI only quantitatively. Additional qualitative and mixed-method research is necessary to obtain a more precise and comprehensive overall explanation of this situation. Second, this study relies on cross-sectional data, which precludes the verification of causal relationships among variables. Future researchers can collect longitudinal data or use experimental designs to determine the causal effect of DL on TI more accurately. Third, as a result of regional differences in the development of education in China, schools in different areas have reached varying stages of modernization in terms of their management practices. Consequently, future researchers should focus on the different stages of school modernization. By collecting data from other regions in China, we aim to investigate the impact of DL on TI across these various developmental stages in further detail. Such research can enable us to validate our findings in further depth and obtain a more comprehensive understanding of the relationship between DL and TI.

## Data Availability

Publicly available datasets were analyzed in this study. This data can be found here: This article relied on the Teaching and Learning International Survey (TALIS) 2018 database (SPSS_2018_international). The website is: https://www.oecd.org/en/data/datasets/talis-2018-database.html.

## Appendix

**Table A1:** OECD latent variable construct.

**Construct**	**Item wording**	**item coding**
Distributed leadership	How strongly do you agree or disagree with these statements, as applied to this school?	
	This school provides staff members with opportunities to participate actively in school decisions	TT3G48A
	This school provides parents or guardians with opportunities to participate actively in school decisions	TT3G48B
	This school provides students with opportunities to participate actively in school decisions	TT3G48C
	This school features a culture of shared responsibility with respect to school issues	TT3G48D
	This school features a collaborative school culture that is characterized by mutual support	TT3G48E
Teacher autonomy	How strongly do you agree or disagree that you have control over the following areas of your planning and teaching in this < target class>?	
	Determining course content	TT3G40A
	Selecting teaching methods	TT3G40B
	Assessing students' learning	TT3G40C
	Disciplining students	TT3G40D
	Determining the amount of homework to be assigned	TT3G40E
Teacher collaboration	On average, how often do you engage in the following activities in this school?	
	Exchanging teaching materials with colleagues	TT3G33D
	Engaging in discussions concerning the learning development of specific students	TT3G33E
	Working with other teachers at this school to ensure the implementation of common standards for evaluations aimed at assessing student progress	TT3G33F
	Attending team conferences	TT3G33G
Teacher self-efficacy	In your teaching, to what extent can you perform the following activities?	
Self-efficacy in terms of classroom management	Controlling disruptive behavior in the classroom	TT3G34D
	Making expectations regarding student behavior clear	TT3G34F
	Ensuring that students follow classroom rules	TT3G34H
	Calming students who are disruptive or noisy	TT3G34I
Self-efficacy in terms of instruction	Crafting good questions to ask students	TT3G34C
	Employing a variety of assessment strategies	TT3G34J
	Providing alternative explanations, for example when students are confused	TT3G34K
	Varying instructional strategies in the classroom	TT3G34L
Self-efficacy in terms of student engagement	Encouraging students to believe that they can complete their schoolwork well	TT3G34A
	Helping students value learning	TT3G34B
	Motivating students who exhibit low interest in schoolwork	TT3G34E
	Helping students think critically	TT3G34G
Teaching innovation	With regard to your teaching in the < target class>, how often do you engage in the following activities?	
cognitive activation	I present tasks for which there is no obvious solution	TT3G42E
	I assign tasks that require students to think critically	TT3G42F
	I require students to work in small groups to develop a joint solution to a problem or task	TT3G42G
	I ask students to identify procedures for solving complex tasks on their own	TT3G42H
enhanced activities	I assign students projects that require at least 1 week to complete	TT3G42O
	I allow students to use ICT (information and communication technology) for projects or class work	TT3G42P
